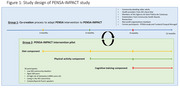# Enhancing access to evidence‐based dementia prevention strategies for vulnerable populations through participatory activities: A feasibility study of a multimodal lifestyle intervention (PENSA‐IMPACT)

**DOI:** 10.1002/alz70858_100842

**Published:** 2025-12-25

**Authors:** Laura Forcano, Ana Aldea, Amaia Ayala‐García, Aida Cuenca‐Royo, Maria Gomis, Natalia Soldevila‐Domenech, Iva Knezevic, Rafael de la Torre

**Affiliations:** ^1^ Integrative Pharmacology and Systems Neurosciences Research Group, Neurosciences Research Program, Hospital del Mar Research Institute, Barcelona, Barcelona, Spain; ^2^ Clinical Research Unit. Hospital del Mar Research Institute, Barcelona, Barcelona, Spain; ^3^ Integrative Pharmacology and Systems Neurosciences Research Group, Neurosciences Research Program, Hospital del Mar Medical Research Institute (IMIM), Barcelona, Barcelona, Spain; ^4^ Barcelonaβeta Brain Research Center (BBRC), Pasqual Maragall Foundation, Barcelona, Barcelona, Spain; ^5^ Integrative Pharmacology and Systems Neurosciences Research Group, Neurosciences Research Program, Hospital del Mar Research Institute, Barcelona, Spain; ^6^ Centro de Investigación Biomédica en Red de Fragilidad y Envejecimiento Saludable (CIBERFES), Instituto de Salud Carlos III, Madrid, Spain; ^7^ Department of Medicine and Life Sciences, University Pompeu Fabra, Barcelona, Spain; ^8^ Centro de Investigación Biomédica en Red Fisiopatología de la Obesidad y la Nutrición (CIBEROBN), Madrid, Spain

## Abstract

**Background:**

Alzheimer's disease (AD) disproportionately affects individuals with low socioeconomic status (SES), who face heightened risks partly due to unhealthy lifestyles and limited access to preventive care. In Spain, over 1.2 million people have dementia, with projections estimating a threefold increase by 2050. Addressing health disparities through adapted interventions is critical. The PENSA project, part of the World‐Wide FINGERS network, has demonstrated efficacy in improving cognitive health through a multimodal lifestyle intervention (MLI) in subjects meeting subjective cognitive decline criteria. However, as commonly observed in clinical trials, the study predominantly attracted volunteers with a median‐high SES. This study aims to adapt the PENSA MLI for low SES older adults using participatory and co‐creation methods.

**Method:**

This feasibility study employs a mixed‐methods design, collecting qualitative data via community dialogues, focus groups, and co‐creation workshops, followed by quantitative assessments from a pilot study. The intervention, incorporating diet, physical activity, and cognitive stimulation, building upon the previous PENSA project's prior work based on the Finger's model, will be adapted in collaboration with end‐users, key stakeholders, and healthcare providers from the Integral Health Area (AIS) Barcelona Litoral Mar, a low SE resources area. Different recruitment strategies will be implemented and assessed to proactively engage individuals from diverse backgrounds, fostering inclusivity in the study sample. A 3‐month qualitative phase will inform a subsequent 3‐month pilot trial.

Thirty community‐dwelling individuals aged ≥60 years with an SES Composite Scale score <16, cognitive performance at or slightly below the expected level (MoCA scores ≥24 points) and a LIBRA score ≥5 will be recruited using snowball sampling and traditional healthcare invitations. Feasibility will be evaluated through participation rates, adherence, satisfaction, and health outcomes.

**Result:**

The study will provide insights into the feasibility of adapting an evidence‐based MLI for low SES populations. Expected outcomes include high participation rates, acceptability, and improved health behaviors.

**Conclusion:**

The PENSA‐IMPACT study seeks to reduce dementia risk in low SES populations by providing an adapted, culturally relevant intervention. If successful, it could inform future escalable interventions to promote equity in dementia prevention.